# GSTO1-1 plays a pro-inflammatory role in models of inflammation, colitis and obesity

**DOI:** 10.1038/s41598-017-17861-6

**Published:** 2017-12-19

**Authors:** Deepthi Menon, Ashlee Innes, Aaron J. Oakley, Jane E. Dahlstrom, Lora M. Jensen, Anne Brüstle, Padmaja Tummala, Melissa Rooke, Marco G. Casarotto, Jonathan B. Baell, Nghi Nguyen, Yiyue Xie, Matthew Cuellar, Jessica Strasser, Jayme L. Dahlin, Michael A. Walters, Gaetan Burgio, Luke A. J. O’Neill, Philip G. Board

**Affiliations:** 1 John Curtin School of Medical Research, Australian National University, Canberra, ACT 2600 Australia; 2 School of Biochemistry and Immunology, Trinity Biomedical Sciences Institute, Trinity College Dublin, Dublin 2, Ireland; 3 School of Chemistry, University of Wollongong, Wollongong, NSW 2522 Australia; 4 ACT Pathology and ANU Medical School, The Canberra Hospital, Garran, ACT 2605 Australia; 5 Monash Institute of Pharmaceutical Sciences, Monash University, Parkville, Victoria, 3052 Australia; 6 Institute for Therapeutics Discovery and Development, University of Minnesota, Minneapolis, MN USA; 7 Department of Pathology, Brigham and Women’s Hospital, Boston, MA USA; 8 School of Pharmaceutical Sciences, Nanjing Tech University, Nanjing, 211816 People’s Republic of China

## Abstract

Glutathione transferase Omega 1 (GSTO1-1) is an atypical GST reported to play a pro-inflammatory role in response to LPS. Here we show that genetic knockout of *Gsto1* alters the response of mice to three distinct inflammatory disease models. GSTO1-1 deficiency ameliorates the inflammatory response stimulated by LPS and attenuates the inflammatory impact of a high fat diet on glucose tolerance and insulin resistance. In contrast, GSTO1-1 deficient mice show a more severe inflammatory response and increased escape of bacteria from the colon into the lymphatic system in a dextran sodium sulfate mediated model of inflammatory bowel disease. These responses are similar to those of TLR4 and MyD88 deficient mice in these models and confirm that GSTO1-1 is critical for a TLR4-like pro-inflammatory response *in vivo*. In wild-type mice, we show that a small molecule inhibitor that covalently binds in the active site of GSTO1-1 can be used to ameliorate the inflammatory response to LPS. Our findings demonstrate the potential therapeutic utility of GSTO1-1 inhibitors in the modulation of inflammation and suggest their possible application in the treatment of a range of inflammatory conditions.

## Introduction

Omega class glutathione transferases (GSTOs) are members of the cytosolic glutathione transferase (GST) super family but have distinct structural and functional attributes that allow them to perform novel roles that are unrelated to the functions of other GSTs^1,2^. While Omega class GSTs have been found to be highly expressed in some tumours, and have been proposed as an anti-cancer drug target, other studies have implicated Omega GSTs as determinants of the age at onset of Alzheimer’s disease and have suggested a pro- inflammatory role for GSTO1-1 in Toll-like receptor 4 (TLR4) linked inflammation^2–6^.

The Omega class GSTs are distinct from the other GST classes because of their active site cysteine residue and their capacity to catalyse several redox reactions that are not catalysed by most other GST isoenzymes^1,2^. GSTO1-1 has been shown to catalyse glutaredoxin (thioltransferase) reactions^1^ as well as the reduction of methylated arsenic species^7,8^, dehydroascorbic acid^1,7^ and S-phenacylglutathiones^9^. In a recent development, our studies have shown that GSTO1-1 can also catalyse the deglutathionylation of proteins^10^. This may be its most physiologically significant role since glutathionylation has been shown to regulate the activity of a varied array of proteins and it has been proposed as a mechanism for the regulation of signaling pathways^10–12^.

In mammals, the initial response to invading pathogens is mounted by the innate immune system. Immune cells such as macrophages, neutrophils and dendritic cells express receptors on their cell surface that recognize a range pathogen components collectively termed pathogen-associated molecular patterns (PAMPS)^13,14^. The response of immune cells to PAMPS can involve the production of reactive oxygen species (ROS), inflammatory cytokines and changes in cellular metabolism to increase glycolysis and decrease oxidative phosphorylation^15,16^. The excessive production of ROS in the inflammatory response can cause tissue damage that is a critical factor in sepsis and the progression of inflammatory diseases^17^. Recent studies have implicated GSTO1-1 in a critical role in the pro-inflammatory response of macrophages to bacterial lipopolysaccharide (LPS) that is mediated through TLR4^6,18^. In those investigations GSTO1-1 expression was knocked down in mouse J774.1 A macrophages by shRNA. The GSTO1-1 deficient cells failed to up-regulate NADPH oxidase 1 expression and produce ROS after LPS stimulation^6^. In metabolic studies, GSTO1-1 deficient macrophage cells stimulated with LPS did not produce excess lactate or dephosphorylate adenosine monophosphate kinase (AMPK) a key metabolic stress regulator^18^. Unlike normal macrophages challenged with LPS, GSTO1-1 deficient macrophages did not accumulate succinate or stabilize HIF1α, typical responses that play significant roles in maintaining the pro-inflammatory state of activated macrophages^16,18^.

To investigate the role of GSTO1-1 in inflammatory pathways *in vivo* we have characterized *Gsto1* knockout mice and investigated their response to several inflammatory disease models, including inflammatory shock from LPS, dextran sodium sulfate mediated colitis and a pro-inflammatory high fat diet. We also investigated the interaction of a small molecule inhibitor with GSTO1-1 and demonstrated that it attenuated the inflammatory response of mice to LPS. Overall these studies indicate that GSTO1-1 plays a pro-inflammatory role in the innate immune system and our identification of the mechanism of action of a small molecule inhibitor and demonstration of its capacity to ameliorate LPS mediated inflammatory shock in mice validates GSTO1-1 as a new target and paves the way for the further development of novel anti-inflammatory drugs.

## Results

### Characterizing GSTO1-1 deficiency in Gsto1^−/−^ mice

**Figure 1:**
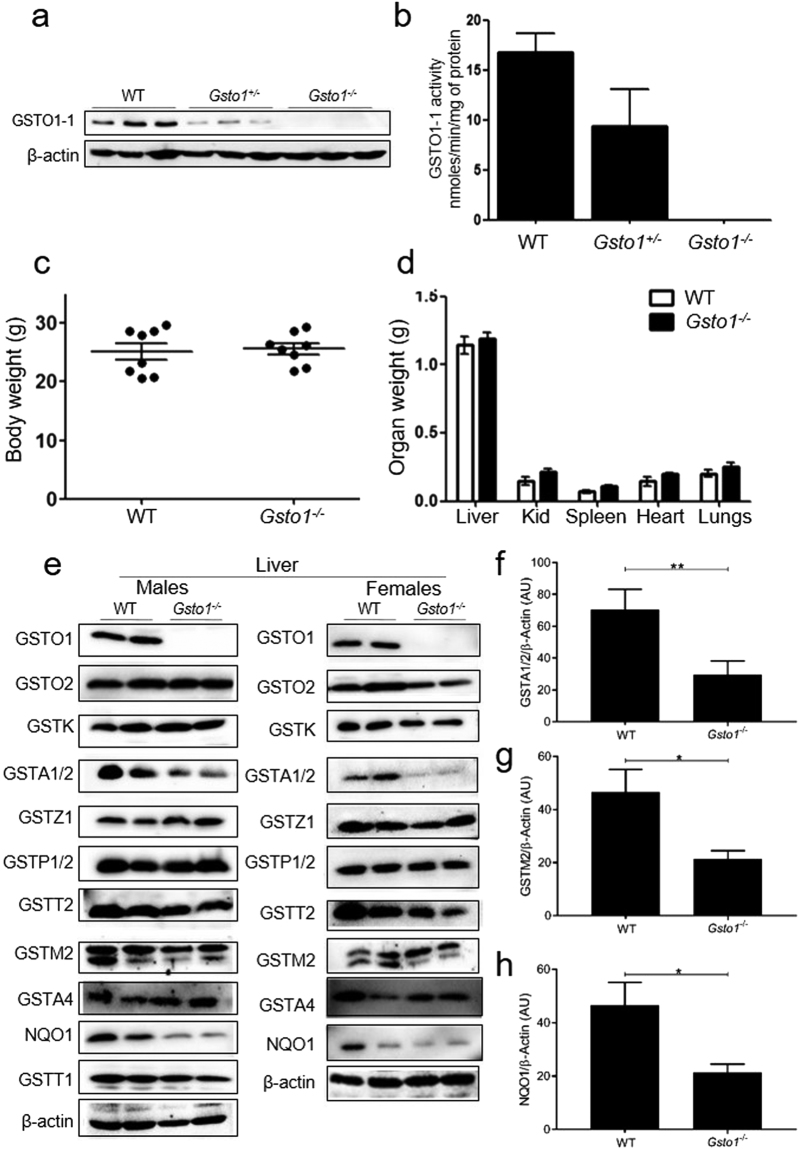
The characteristics of GSTO1-1 deficiency in mice. (**a**) An immunoblot showing the absence of GSTO1-1 protein in the liver of *Gsto1* ^−/−^ mice. (**b**) GSTO1-1 enzymatic activity with 4NPG as a substrate is absent in the liver of *Gsto1* ^−/−^ mice (n = 7). (**c**) The body weight of 8 weeks old male *Gsto1* ^+/+^ and *Gsto1* ^−/−^ mice, (Mean ± SE n = 8). (**d**) There were no differences in the weights of the major organs from *Gsto1* ^+/+^ and *Gsto1* ^−/−^ mice (Mean ± SE n = 8). (**e**) Immuno blots of GST proteins and NQO1 in *Gsto1* ^+/+^ and *Gsto1* ^−/−^ mice. (**f**–**h**) Quantitative comparison of GSTA1/2, GSTM2, and NQO1levels normalized to actin (Mean ± SE n = 4). (*p < 0.05, **p,0.01).

Immunoblotting of liver extracts revealed that GSTO1-1 was absent in *Gsto1* ^−/−^ mice while heterozygous (*Gsto1* ^+/−^) mice expressed approximately half the amount detected in wildtype mice (Fig. 1a). To further confirm that the expression of GSTO1-1 was completely absent, the enzyme activity of GSTO1-1 with the specific substrate 4-NPG was found to be undetectable in the liver of *Gsto1* ^−/−^ mice (Fig. 1b) while heterozygotes exhibited half the activity measured in wildtype mice. The general characteristics of *Gsto1* ^−/−^ mice including body weight and organ weights were determined to evaluate whether GSTO1-1 deficiency caused any developmental/growth abnormalities. As shown in Fig. 1c, the total body weight of *Gsto1* ^−/−^ mice (8 weeks of age, males) clustered well within the limits set by wildtype littermates. Furthermore, the major organs were visually normal and the organ weights did not differ from the wild-type controls (Fig. 1d), suggesting there are no major organ deformities in the *Gsto1* ^−/−^ mice under normal physiological conditions. Histological examination of liver, kidney, heart and lungs by light microscopy of H&E sections did not reveal any significant differences between wildtype and Gsto1^−/−^ mice (data not shown). The mean values for serum alanine aminotransferase (ALT) (WT 2.9 ± 0.3U vs KO 4.3 ± 1.2U) and creatinine (WT 74.5 ± 2.7 µg/dL vs KO 80.2 ± 3.7 µg/dL) in 6 wild-type and 6 *Gsto1* ^−/−^ mice did not differ significantly supporting the view that liver and kidney function were normal.

### Expression profile of glutathione transferases in Gsto1^−/−^ mice

Previously studies of *Gstz1* knockout mice revealed an increase in the expression levels of other GSTs in response to the increased oxidative stress in mice that are deficient in glutathione transferase Zeta^19^. Given the reduction reactions catalysed by GSTO1-1 we determined whether *Gsto1* ^−/−^ mice showed a compensating increase in the expression of other GSTs and redox sensitive enzymes. Liver lysates were extracted and cytosolic proteins were separated by SDS PAGE and immunoblotted with specific antiserum. These blots revealed a significant (p < 0.05) decrease in the expression of GSTA1/2, and GSTM2 (lower band) (Fig. 1e–g). However, the expression of GST subunits such as GSTP1, GSTK1, GSTO2, GSTT1 and GSTT2 in *Gsto1* ^−/−^ mice was the same as that in the wildtype mice. The expression levels of NAD(P)H: quinone oxidoreductase 1 (NQO1), considered a good indicator of oxidative stress in cells and a target of Nrf2 redox dependent regulation, was also found to be significantly (p < 0.05) lower in the *Gsto1* ^−/−^ mice (Fig. 1e,h). The differences observed in GST and NQO1 expression levels between *Gsto1* ^−/−^ and wild-type mice were similar in male and female mice. The results suggest that deficiency of GSTO1-1 does not result in the compensatory induction of other GSTs and the diminished expression of NQO1 in *Gsto1* ^−/−^ mice further suggests that GSTO1-1 deficiency does not give rise to increased oxidative stress.

### Hematological changes associated with GSTO1-1 deficiency

To further characterize the impact of GSTO1-1 deficiency, the hematological profile of *Gsto1* ^−/−^ mice was evaluated in 8-week-old mice (n = 10) (Table 1). There were no significant differences in the red blood cell indices of wildtype and *Gsto1* ^−/−^ mice. Although WBCs including basophils, neutrophils and lymphocytes were similar in wild-type and *Gsto1* ^−/−^ mice, the monocyte and eosinophil counts of *Gsto1* ^−/−^ mice were significantly (P ≤ 0.05) lower than their wild-type counterparts. Monocytes differentiate into resident macrophages in tissues, and exacerbate inflammation^20^. The lower monocyte and eosinophil counts in the *Gsto1* ^−/−^ mice under normal physiological conditions suggests that there may be a defect in the differentiation of myeloid cells or possibly a change in the turnover or sequestration macrophages that warrants further investigation.

**Table 1:** Hematological evaluation of wild-type and Gsto1^−/−^ mice.

	Wild-type	*Gsto1* ^−/−^
RBC (x10E06 cells/µL)	11.7 ± 0.23	12.1 ± 0.17
HGB (g/L)	210.5 ± 4.29	211.4 ± 3.95
HCT	0.5 ± 0.01	0.5 ± 0.01
MCV(fL)	45.8 ± 0.5	45.3 ± 0.36
Platelets (x10E03 cells/µL)	923.6 ± 91.65	965 ± 73.27
WBC (x10E03 cells/µL)	10.3 ± 1.06	9.8 ± 0.44
Neutrophils (x10E03 cells/µL)	1.5 ± 0.26	1.7 ± 0.35
Lymphocytes (x10E03 cells/µL)	5.8 ± 1.38	4.6 ± 1.12
Monocytes (x10E03 cells/µL)*	0.3 ± 0.06	0.18 ± 0.03
Eosinophils (x10E03 cells/µL)*	0.2 ± 0.04	0.1 ± 0.02
Basophils (x10E03 cells/µL)	0.02 ± 0.01	0.04 ± 0.01

Mean ± SE n = 10 (*p ≤ 0.05).

### Gsto1^−/−^ mice are resistant to LPS induced inflammatory shock

**Figure 2:**
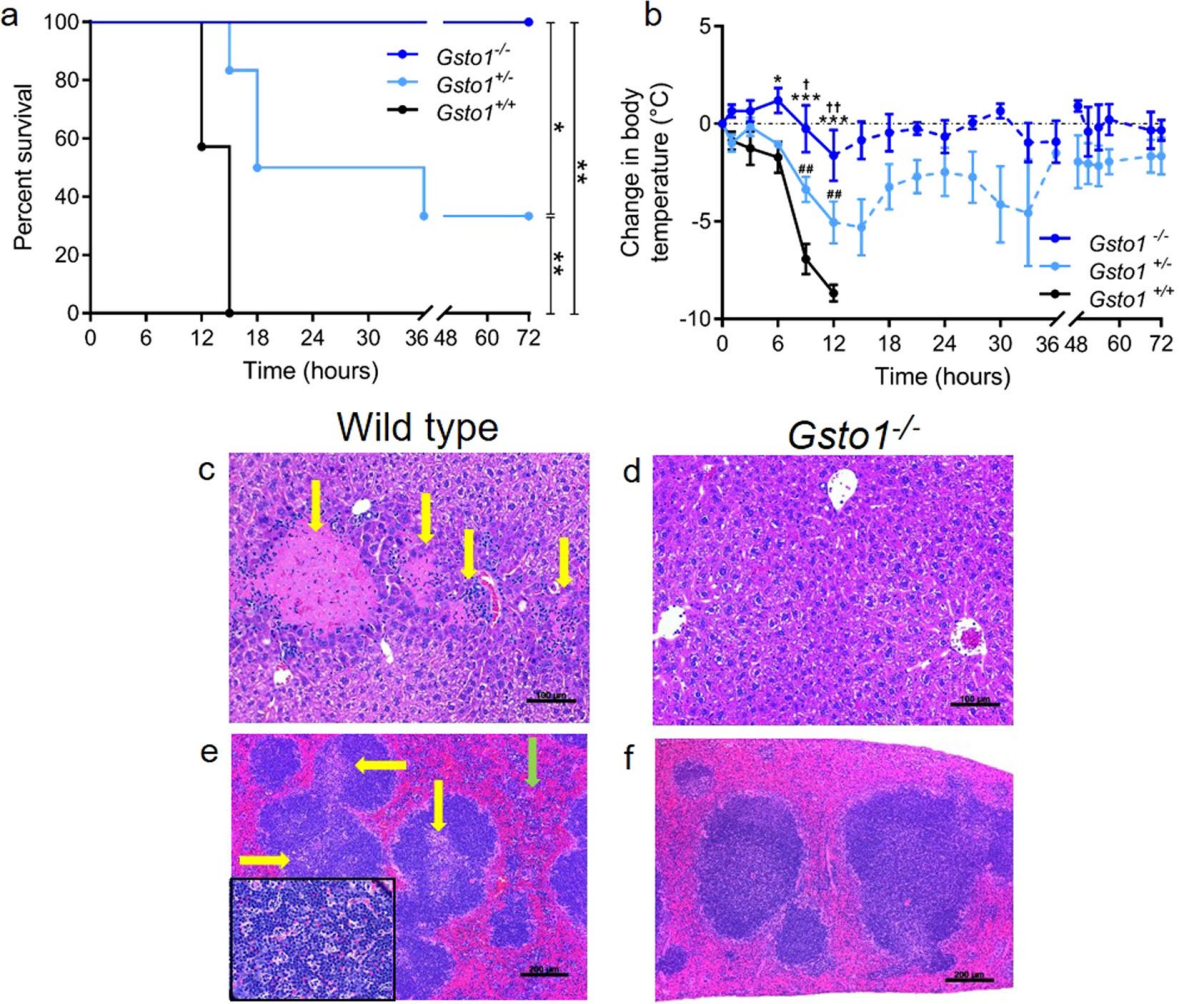
Response of *Gsto1* ^−/−^ mice to LPS. (**a**) Kaplan-Meyer plot showing survival of male mice treated with 10 mg/kg LPS (*p < 0.05, **p,0.01). (**b**) Body temperature of mice treated with LPS, Mean ± SE *Gsto1* ^+/+^ n = 7, *Gsto1* ^+/−^ n = 6, *Gsto1* ^−/−^ n = 5 (*p < 0.05, **p,0.01, ***p < 0.001).The significance of different comparisons are indicated as (* −/− vs +/+); († −/− vs +/−); (# +/− vs +/+). The dashed lines indicate times when there were too few surviving wild type mice to allow statistical comparison. Mice were ethically euthanazed if the decrease in temperature exceeded 10 °C or they reached a predetermined morbidity score. This experiment is representative of several experiments undertaken with different LPS concentrations (see Supplementary Fig. S1 for female mice). (**c**) liver of wild-type mouse treated with 50 mg/kg LPS for 3 hours demonstrating multiple foci of acute inflammation with necrosis (**c)** yellow arrows) which was absent in the liver of *Gsto1* ^−/−^ mice receiving the same LPS treatment (**d**). (**e**) spleen histology of wild-type mouse treated with 50 mg/kg LPS for 6 hours demonstrating expansion of the white pulp due to accumulation of tingible body macrophages (yellow arrows) and increased inflammatory cells in the red pulp (green arrow), insert shows tingible body macrophages, ×630. (**f**) spleen of wild-type mouse treated with 50 mg/kg LPS for 6 hours with normal appearing red and white pulp.

In previous studies we found that GSTO1-1 deficient macrophages do not develop a pro-inflammatory phenotype when challenged with LPS^6,18^. In the present *in vivo* study, LPS responsiveness was investigated *in vivo* by monitoring the body temperature and survival of age matched male *Gsto1* ^−/−^, *Gsto1* ^+/−^ and wild-type *Gsto1* ^+/+^ mice after an *ip* injection of LPS. At a dose of 10 mg LPS/kg the wild-type mice showed marked hypothermia and all wild-type mice reached an adverse clinical score and ethical surrogate marker of death within 15 hours post LPS injection. In contrast the knock out mice showed high resistance to LPS induced inflammatory shock with minimal changes in body temperature and 100% survival (Fig. 2a,b KO vs WT p < 0.001). Interestingly, although the *Gsto1* ^+/−^ mice responded adversely to LPS they were less sensitive than wild-type mice and presented an intermediate response in their body temperature (Het vs WT p < 0.01). The intermediate response of *Gsto1* ^+/−^ mice suggests that even partial deficiency of GSTO1-1 can protect against LPS mediated inflammatory shock. The similar response of female mice is shown in Supplementary Fig. S1.

### Pathological changes associated with GSTO1-1 deficiency and LPS exposure

Histological examination of the liver from mice treated with 50 mg LPS/kg (n = 3/time period) resulted in patchy acute hepatitis with focal necrosis after 1 hour in wild-type mice. In contrast this pathology was completely absent in *Gsto1* ^−/−^ mice up to 12 hours post LPS (shown after 3 hours in Fig. 2c,d). Histological examination of the spleen from mice treated with 50 mg LPS/kg (n = 3/time period) revealed a variable number of tingible body macrophages, mainly in the white pulp areas, in wild type mice at 1, 3 and 6 hours post LPS (Fig. 2e). In the red pulp, areas of acute inflammation were apparent in wild-type mice 3 hours post LPS treatment (Fig. 2e). This was in contrast to the *Gsto1* ^−/−^ mice that presented normal spleen histology for at least 6 hours (Fig. 2f).

### Attenuated pro-inflammatory cytokine expression in LPS treated Gsto1^−/−^ mice

**Figure 3:**
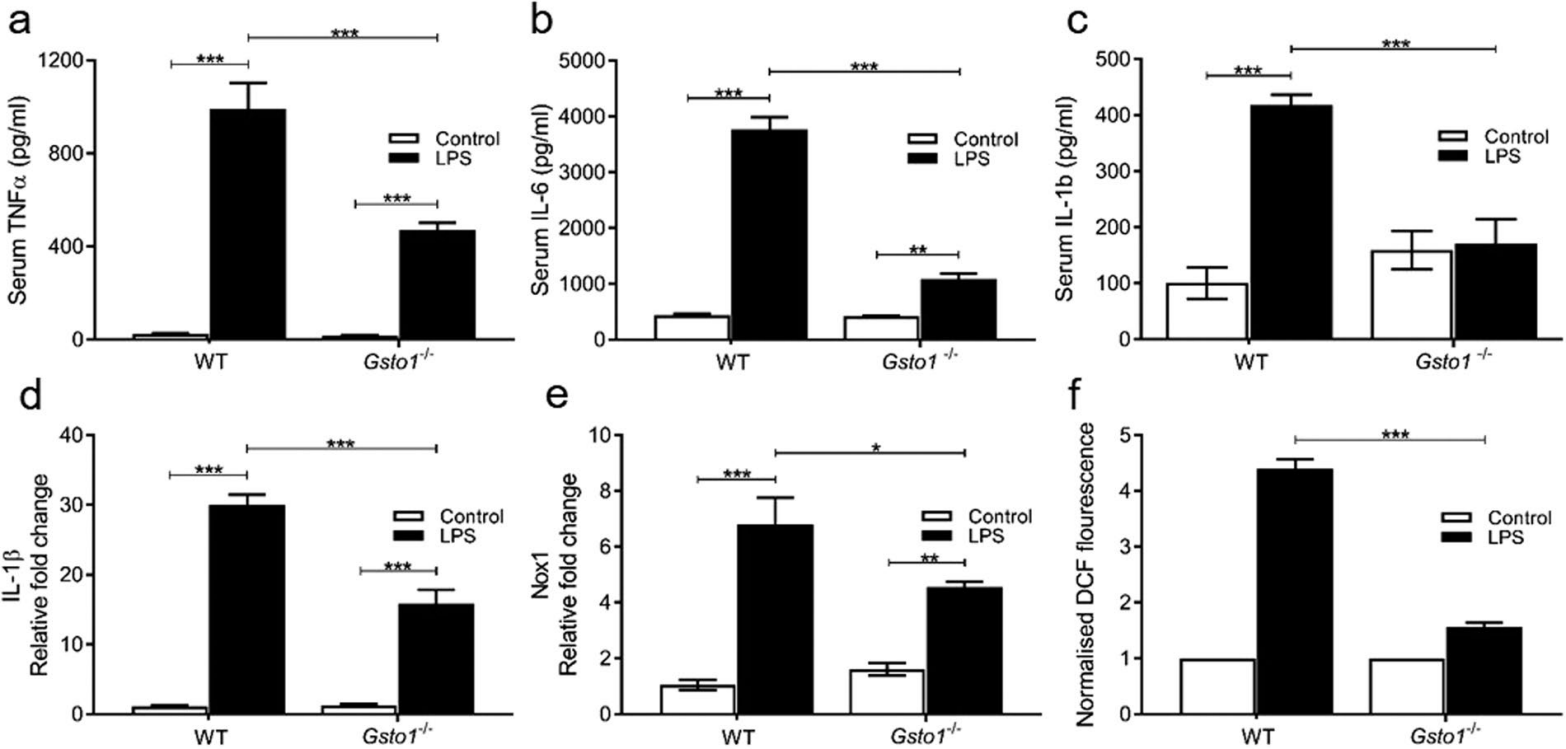
The diminished inflammatory response of *Gsto1* ^−/−^ mice to LPS at 50 mg/kg for 30 minutes, n = 5 or 6. (**a**) Serum TNF. (**b**) Serum IL-6. (**c**) Serum IL-1β. (**d**) Spleen IL-1β RNA expression. (**e**) Spleen NADPH oxidase 1(Nox1) RNA expression. (**f**) ROS production from BMDM treated with 200 ng/mL LPS for 1 hour. Data shown as Mean ± SE n = 5 or 6, *P < 0.05, **P < 0.01, ***P < 0.001.

The response of *Gsto1* ^−/−^ mice to LPS was further examined by determining the serum cytokine levels in mice injected with 50 mg LPS/kg. Wild-type mice produced significantly elevated levels of cytokines TNF-α, IL-6 and IL-1β 30 minutes after injection with LPS (Fig. 3a–c). In contrast, the *Gsto1* ^−/−^ mice were less responsive to LPS and produced significantly lower levels of all three cytokines when compared with wild type mice (Fig. 3a–c). The change in serum cytokine levels in the wild-type mice was similar to the trend in previously published studies^21,22^. IL-1β expression in the spleen was also significantly (p < 0.001) suppressed in *Gsto1* ^−/−^ mice relative to the response of wild-type mice (Fig. 3d). A similar suppression of IL-1β expression was also observed in the liver 12 hours after LPS treatment (data not shown). The increased survival of *Gsto1* ^−/−^ mice treated with LPS correlates well with their suppressed pro-inflammatory cytokine expression and the lack of liver necrosis.

### LPS induced NADPH oxidase 1 transcript levels were attenuated in Gsto1^−/−^ mice

Knockdown of GSTO1-1 in J774.1 A macrophages has been shown to attenuate the induction of NOX1 and the production of ROS by LPS^6^. In this study NOX1 transcription was significantly (p < 0.001) induced in the spleen of wild-type mice 3 hours after injection with 50 mg LPS/kg. Although NOX1 was also induced in LPS treated *Gsto1* ^−/−^ mice (Fig. 3e), the levels were significantly (p < 0.05) less than those achieved in wild-type mice. In order to examine the direct of impact GSTO1-1 deficiency on ROS expression we determined ROS levels in bone marrow derived macrophage (BMDM) cultures after LPS treatment (200 ng/mL for 1 hour). The results indicated a highly significant (p < 0.001) attenuation of ROS production in GSTO1-1 deficient BMDMs (Fig. 3f). This confirmed, in *Gsto1* ^−/−^ mice, the significant contribution of GSTO1-1 to LPS mediated NOX1 induction and ROS generation previously reported in J774.1 A macrophages^6^.

### Gsto1^−/−^ mice are less susceptible to HFD-induced obesity and inflammation

**Figure 4:**
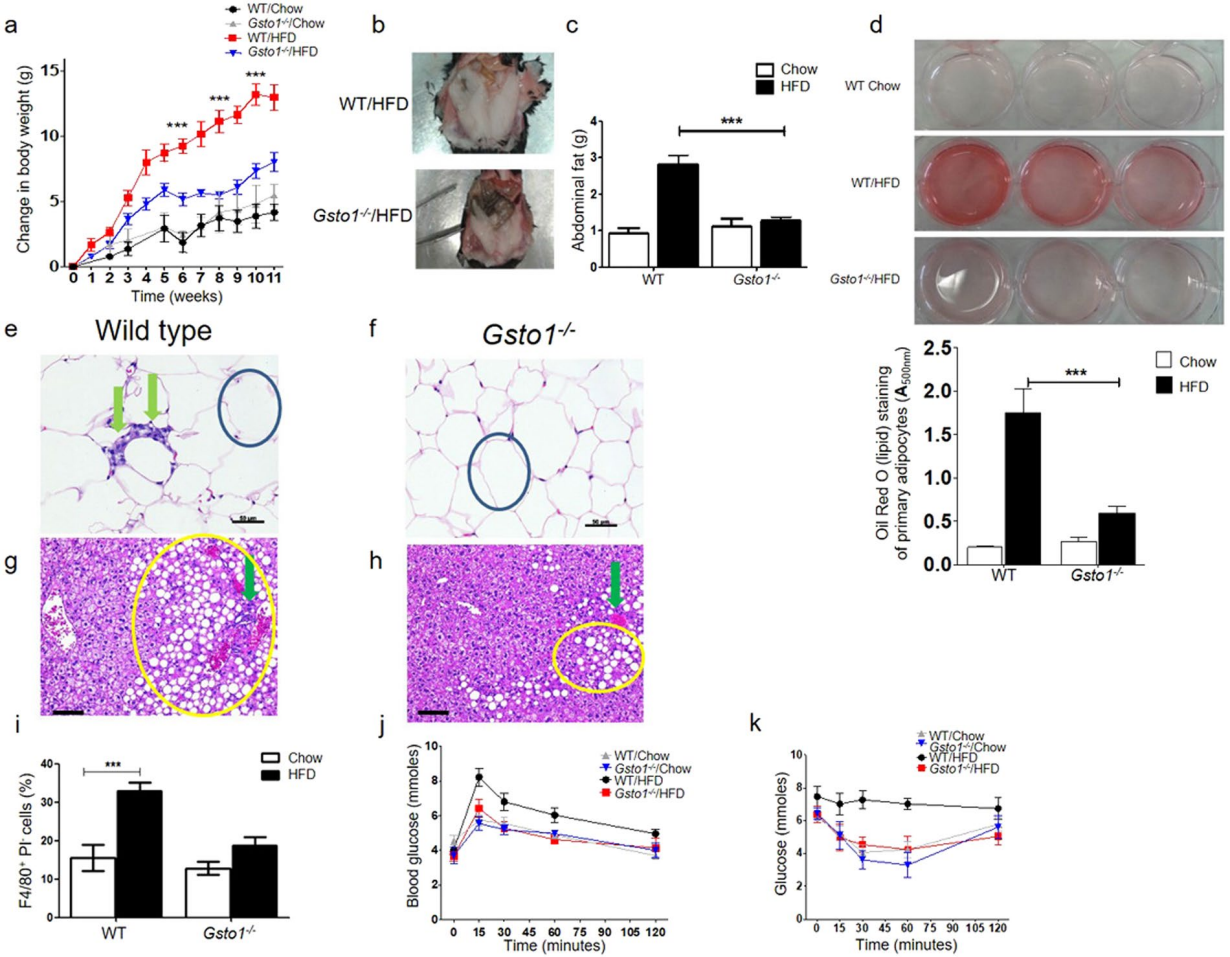
Response of *Gsto1* ^−/−^ mice to a high fat diet. (**a**) Weekly weight gain on high fat and normal chow diets (Mean ± SE n = 9). (**b**) Abdominal fat pads from wild type and *Gsto1* ^−/−^ mice after 13 weeks on a high fat diet. (**c**) Weight of abdominal fat after 13 weeks on a high fat diet (p < 0.001 Mean ± SE n = 9). (**d**) Lipid accumulation in isolated adipocytes from *Gsto1* ^−/−^/HFD mice was significantly less than wildtype/HFD mice as quantified by oil red O staining (13 weeks on HFD, Mean ± SE n = 9). After 13 weeks on a high fat diet inflammation (green arrows) is seen in adipose tissue in wild-type mice (**e**) and was often not seen in *Gsto1* ^−/−^ mice (**f**) (circle indicates an adipocyte). More steatosis (yellow circled areas) is present in the livers of the wild-type mice (**g**) compared to *Gsto1* ^−/−^ mice (**h**) after 13 weeks on a high fat diet (green arrow indicates a portal tract). (**i**) The number of adipose tissue associated macrophages is significantly increased in wild type mice receiving a high fat diet (p < 0.001 Mean ± SE n = 9). (**j**) Blood glucose response to a glucose load in mice fed a high fat diet (Mean ± SE n = 9). (**k**) Blood glucose response to insulin in mice fed a high fat diet (Mean ± SE n = 9).

TLR4 and MyD88 play prominent roles in signaling that supports low level inflammation in obesity. Deficiency of either of these proteins attenuates obesity and the metabolic changes that occur in response to a high fat diet (HFD)^23,24^. Since GSTO1-1 appears to play a role in the TLR4 and MyD88 inflammatory pathway, we investigated the response of *Gsto1* ^−/−^ mice to a high fat diet. *Gsto1* ^−/−^ mice maintained on a diet containing 23% fat for 13 weeks were phenotypically similar to MyD88^−/−^ and TLR4^−/−^ mice fed a similar diet^23,24^. The *Gsto1* ^−/−^ mice failed to gain additional weight when fed a HFD while the wild-type mice gained 1-2 g per week more than chow fed wild-type controls (Fig. 4a). On dissection, the amount of abdominal fat in wild-type mice fed the high fat diet was significantly greater than that found in the *Gsto1* ^−/−^ mice (p < 0.001) (Fig. 4b,c). The lipid content in isolated adipocytes was determined by oil red O staining. As shown in Fig. 4d, the amount of accumulated lipids in high fat diet fed Gsto1^−/−^ mice was significantly less (p < 0.001) than that occurring in their wildtype counterparts. Histological examination of the abdominal adipose tissue revealed increased inflammation in wild-type mice compared with *Gsto1* ^−/−^ mice (Fig. 4e,f). Furthermore, the number of infiltrating activated macrophages (F4/80+) isolated from the abdominal white adipose tissue was remarkably higher in the wild-type mice compared to the *Gsto1* ^−/−^ mice (Fig. 4i). Examination of the liver revealed more steatosis and acute inflammation in wild-type mice fed a HFD compared to *Gsto1* ^−/−^ mice (Fig. 4g,h). Over all this suggests a higher level of inflammation in the wild-type mice. As expected, wild-type mice fed a HFD acquired insulin resistance over the period of the diet and were unable to utilize glucose as rapidly as the chow fed mice (Fig. 4j,k). In contrast the HFD/*Gsto1* ^−/−^ mice remained responsive to insulin suggesting that they are resistant to HFD induced metabolic distress (Fig. 4j,k).

### Gsto1^−/−^ mice show increased susceptibility to dextran sodium sulfate induced colitis

**Figure 5:**
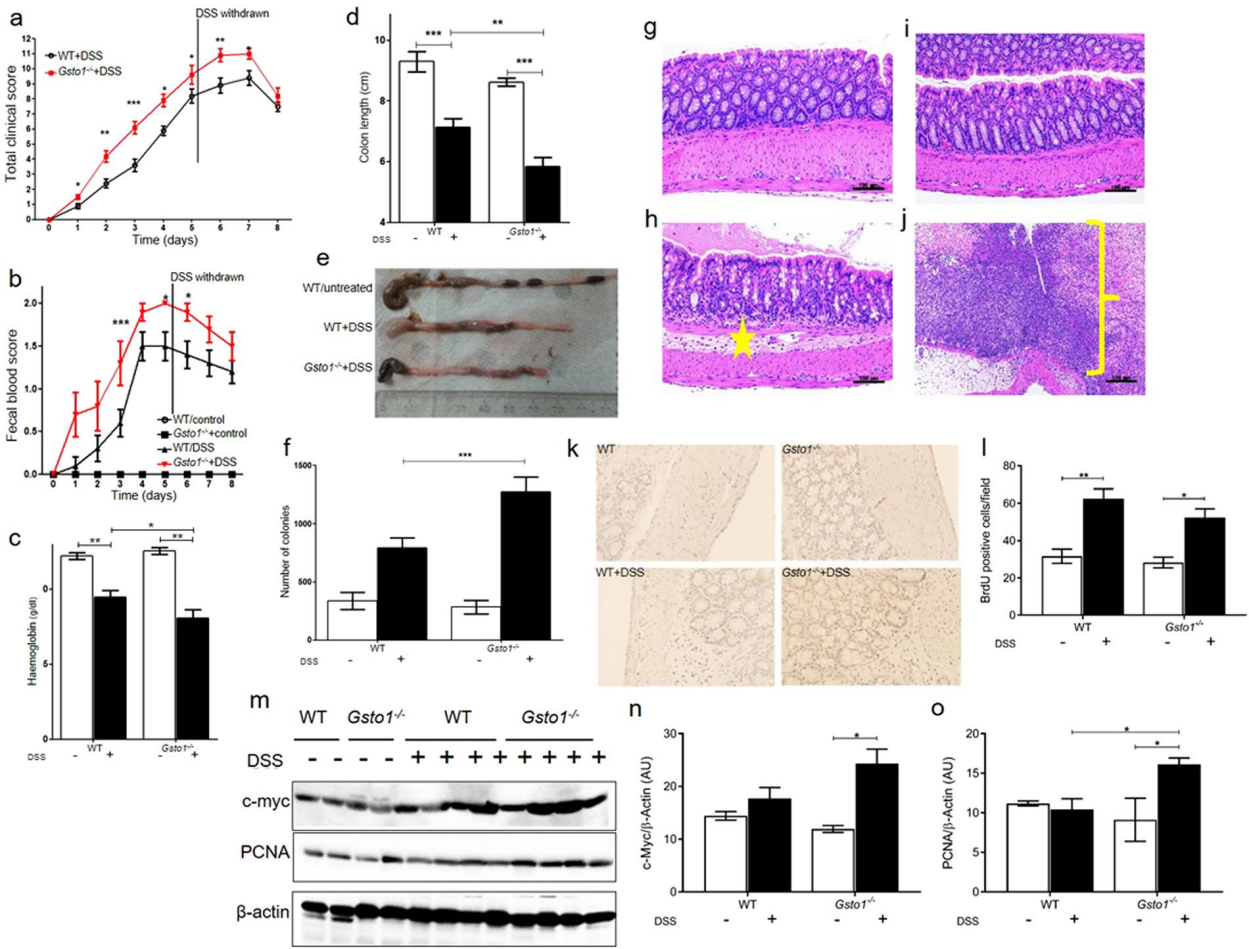
Sensitivity of mice to DSS mediated colitis. (**a**) Total clinical score comprising weight, stool consistency, blood at orifice and fecal blood, after DSS treatment, Mean ± SE n = 10. (**b**) fecal blood score after DSS treatment, Mean ± SE n = 10. (**c**) Blood hemoglobin after DSS treatment was significantly lower in *Gsto1* ^−/−^ mice than in wildtype mice, (p < 0.05 Mean ± SE n = 10). (**d**,**e**) Colon length after DSS treatment, Mean ± SE n = 10. (**f**) Number of bacterial colonies escaping to the mesenteric lymph gland after DSS treatment is significantly greater in *Gsto1* ^−/−^ mice, (p < 0.001 Mean ± SE n = 10). (**g** and **h**) show sections of distal colon from water and DSS fed wild-type mice. The wild-type mice fed water showed normal colonic mucosa (**g**), while the DSS fed mice showed mild acute colitis confined to the mucosa and submucosa without ulceration (star symbol indicates the submucosal oedema and inflammatory cell infiltrate) (**h**). (**I** and **J**) show sections of distal colon from water (**I**) and DSS fed *Gsto1* ^−/−^ mice (**j**). While the distal colon from the water fed mice is normal the DSS fed *Gsto1* ^−/−^ mice showed severe acute colitis with transmural inflammation with mucosal ulceration (yellow brackets shows absence of mucosal epithelium and pus indicative of acute ulceration). (**k**,**l**) BrdU incorporation into DNA in distal colonic sections was assessed immunohistochemically to evaluate the extent of epithelial cell proliferation in response to DSS (Mean ± SE n = 6). (**m**–**o**) Cell proliferation was also assessed by immunostaining c-myc (**n**) and PCNA (**o**) in protein blots from the distal colon (Mean ± SE n = 4). M is a representative blot from 2 replicates. *P < 0.05, **P < 0.01, ***P < 0.001.

In mice, ingestion of dextran sodium sulfate (DSS) induces colitis and a major inflammatory response to the escape of microbial flora from the colon. Since our current experiments have shown that *Gsto1* deficient mice have a defect in LPS stimulated inflammation, we evaluated the development of dextran sulfate mediated colitis in *Gsto1* ^−/−^ mice. Mice were treated with a dose of 3.5% DSS in their drinking water for 5 consecutive days followed by a 3-day period of receiving regular water. Both wild-type and *Gsto1* ^−/−^ mice demonstrated weight loss though the extent of disease progression was significantly exacerbated in the *Gsto1* ^−/−^ mice from day 1. The severity of colitis in these mice was evaluated as a clinical score by summing the percentile of weight loss, fecal blood, stool consistency and occurrence of blood in the anal orifice (Fig. 5a,b). *Gsto1* ^−/−^ mice scored at an average of 10.6/14 while the wild-type mice exhibited a less intense response (8/14) 8 days after the initiation of the experiment. Since the blood loss associated with colitis induces anemia in colitis patients, we determined the blood hemoglobin levels in *Gsto1* ^−/−^ mice. As shown in Fig. 5c, both the wild-type and *Gsto1* ^−/−^ mice treated with DSS presented with a significant drop in their hemoglobin levels (p < 0.01). However, the decrease was more pronounced in the *Gsto1* ^−/−^ mice (p < 0.05), complimenting their higher clinical score. The colonic length was also determined as an additional measure of the severity of the colitis. The colonic length in wild-type and *Gsto1* ^−/−^ mice receiving regular drinking water ranged between 8.2 to 10.5 cm and the two groups were not significantly different. However, the colonic length in DSS-fed mice was significantly shorter (P < 0.001), than the water-fed mice, and the average of the DSS treated *Gsto1* ^−/−^ mice (5.8 ± 0.68 cm) was significantly shorter (P < 0.01) than the average of the DSS treated wild-type mice (7.1 ± 0.6 cm) (Fig. 5d,e).

We next examined the gut flora escaping into the mesenteric lymph nodes as an additional measure of disease severity. Mesenteric lymph nodes that drain the intestine were isolated and cultured *in vitro*. The number of bacterial colonies observed in DSS fed *Gsto1* ^−/−^ mice was significantly higher (P < 0.001) than the number counted in the wild-type mice, suggesting that the disease was more severe in the absence of GSTO1-1 (Fig. 5f). Histological examination of the distal colon did not reveal any abnormalities in the wild-type or *Gsto1* ^−/−^ mice fed water. The DSS treated *Gsto1* ^−/−^ mice developed severe transmural acute colitis with extensive mucosal ulceration while the DSS treated wild-type mice developed much less severe acute colitis without ulceration (Fig. 5g–j). In order to assess the extent of intestinal damage in response to DSS, we examined the rate of proliferation of intestinal epithelial cells using multiple markers. Staining for BrdU positive cells in intestinal sections showed that DSS treatment caused a significant increase in proliferation of epithelial cells along the colon lining in both wild-type and *Gsto1* ^−/−^ mice. However there was no difference in the number of BrdU positive cells in the DSS treated *Gsto1* ^−/−^ mice compared to the wildtype counterparts (Fig. 5k,l). In contrast the proliferation markers c-myc and Proliferating Cell Nuclear Antigen (PCNA) showed significantly increased expression in DSS treated *Gsto1* ^−/−^ mice (Fig. 5m–o). Taken together these data suggest that *Gsto1* ^−/−^ mice are more sensitive to DSS than wild type mice and show increased proliferation of intestinal epithelial cells.

### ML175 covalently labels to the active site cysteine residue (Cys 32)

**Figure 6:**
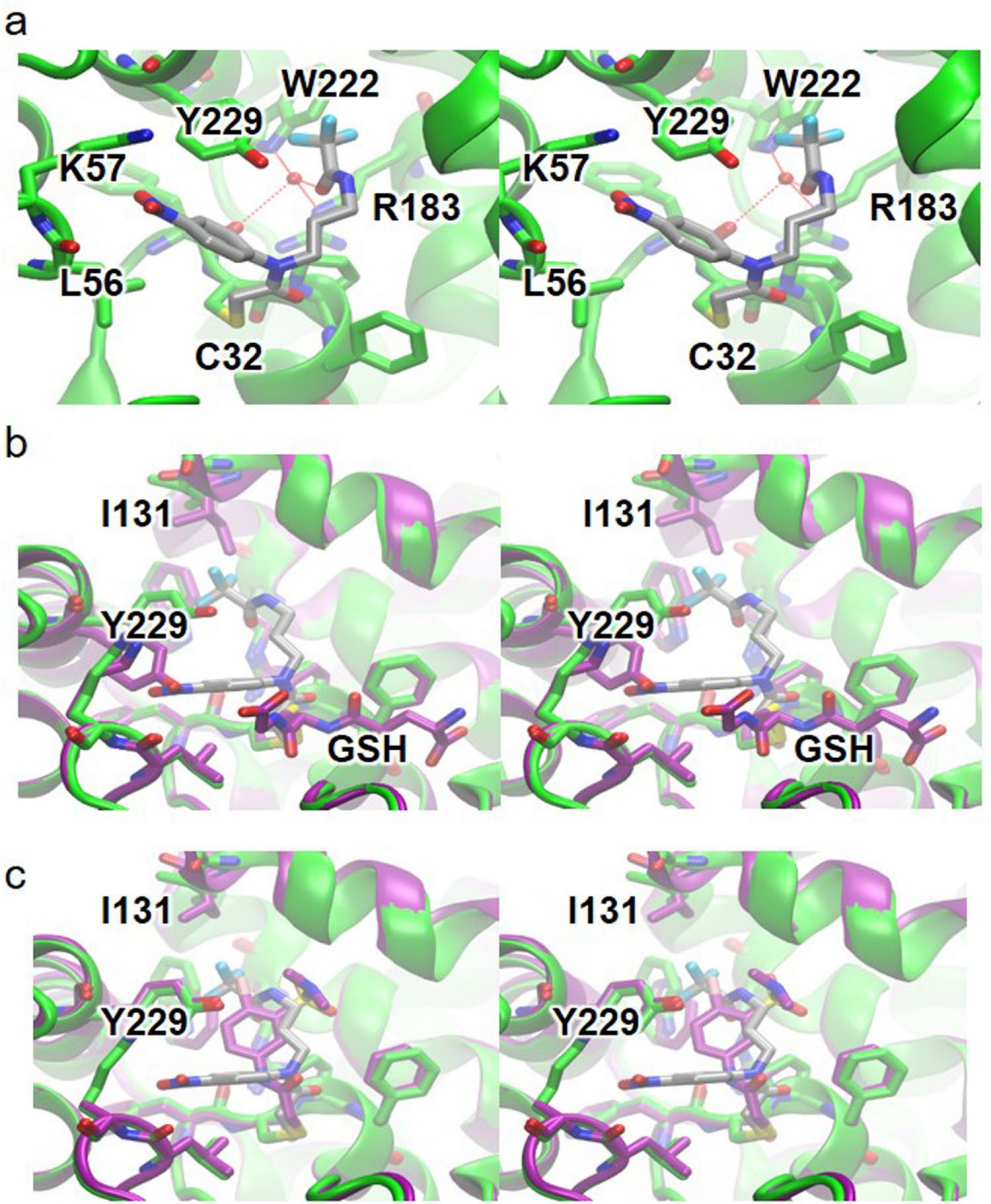
The structure of GSTO1-1 with ML175. (**A**) stereo-diagram showing the model of GSTO1-1 with ML175 bound to C32. ML175 (grey carbon atoms) and surrounding residues (green carbon atoms) are represented in stick form. The backbone of the protein is represented in cartoon form. (**b**) Stereo-diagram showing the active site of GSTO1-1 in complex with ML175 (green) and GSH (purple) superposed. (**c**) stereo diagram showing the active site of GSTO1-1 in complex with ML175 (green) and C1-27 (purple) superposed. The backbones of the proteins are represented in cartoon form.

**Figure 7:**
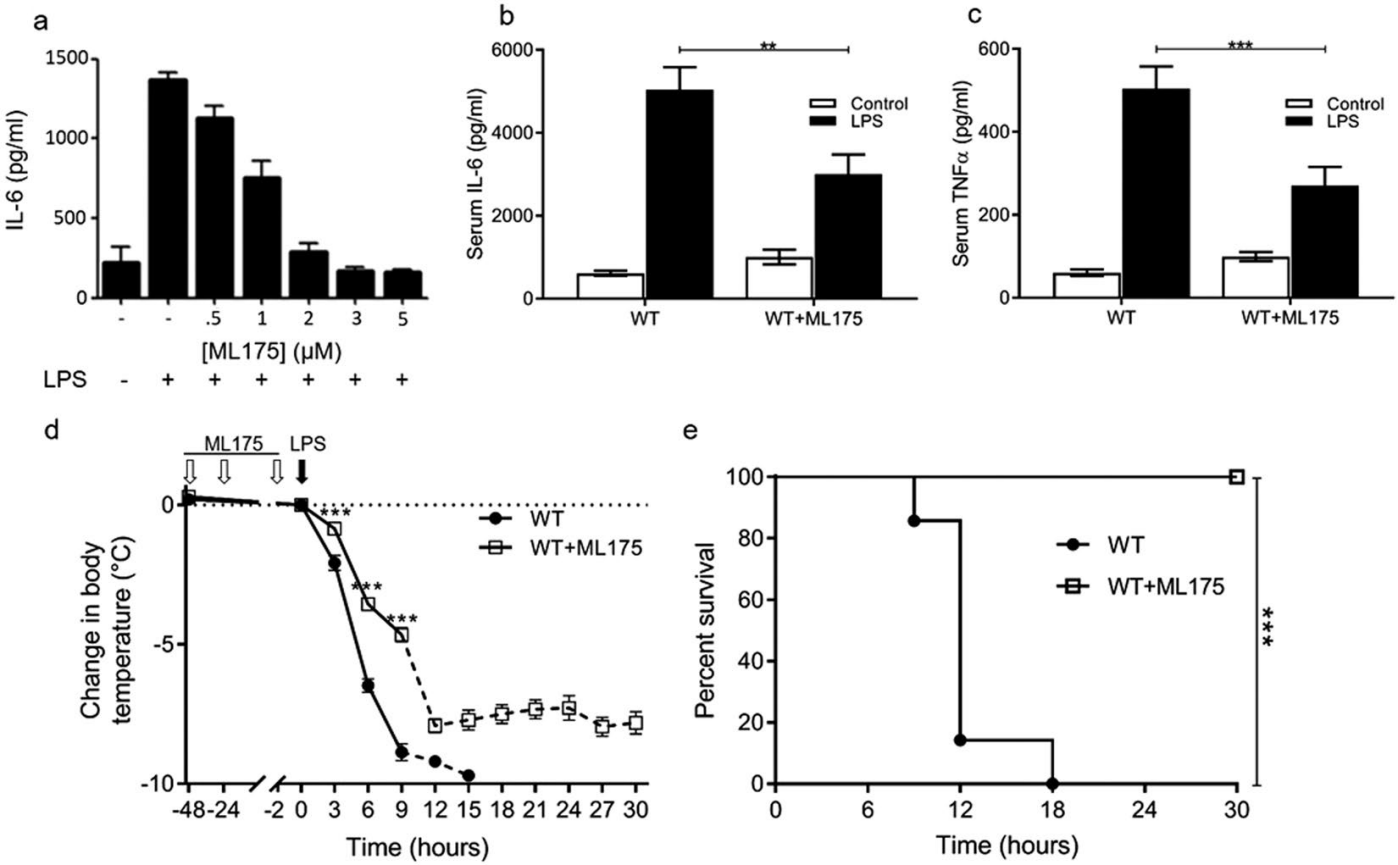
ML175 suppresses the inflammatory response to LPS. (**a**) ML175 dose dependent suppression of IL-6 secretion from LPS treated BMDMs, Mean ± SE n = 3. (**b**) Serum IL-6 in mice treated with LPS (50 mg/kg) and ML175 (10 mg/kg) (Mean ± SE n = 5 or 6, *P < 0.05). (**c**) Serum TNFα in mice treated with LPS (50 mg/kg) and ML175 (20 mg/kg), (Mean ± SE n = 6 **P < 0.01). (**d**) Change in temperature after ip LPS injection (15 mg/kg) and treatment with ML175 10 mg/kg at −48, −24, and −2 hours prior to LPS, Mean ± SE n = 7. The dashed lines indicate times when there were too few surviving wild type mice to allow statistical comparison.

Given the importance of GSTO1-1 in the inflammatory response to LPS and other mouse models of inflammation it is evident that GSTO1-1 is a novel target for the development of new anti-inflammatory drugs. In previous studies we have shown that the GSTO1-1 inhibitor ML175 can block the expression of NOX1 and the generation of ROS in macrophages after treatment with LPS^6^. To examine the mechanism underlying the inhibition by ML175 we solved the crystal structure of GSTO1-1 in complex with ML175. The overall structure of the GSTO1-1 - ML175 complex is essentially the same as the previously published complex with GSH^1^. Superposing the structures results in a RMSD of 0.36 Å over 237 Cα atoms. ML175 binds in the “H-site” of GSTO1-1 and is covalently attached to catalytic residue Cys32. Clear electron density was observed for the nitrophenyl moiety, with weaker density observed for the propyltrifluoroacetamide group. The flexible nature of the propyltrifluoroacetamide group may explain this observation. The chloride atom of ML175 has been displaced by the sulfur atom of C32. The nitrophenyl moiety is oriented toward the side-chain of Lys57, and sits between Leu56 and Tyr229. The propyltrifluoroacetamide moiety lies in an extended conformation oriented toward the back of the “H-site”, contacting residues Pro33, Val127, Ile131 and Trp222 (Fig. 6a). Binding of ML175 induced rotation of residues Tyr229 and Ile131 (Fig. 7b). A water molecule mediates interaction between the side-chains of Arg183, Trp222, the carbonyl oxygen of Phe31, and the carbonyl oxygen of the ML175 propyltrifluoroacetamide moiety (Fig. 6a). The previously reported GSTO1-1 inhibitor C1-27^3^ binds in essentially the same pocket as ML175, and like ML175, covalently modifies Cys32 (Fig. 6c).

### ML175 is not generally thiol-reactive

Given its covalent mechanism of target engagement, we tested ML175 by ALARM NMR, an industry-developed heteronuclear multiple-quantum correlation (HMQC) counter-screen for nonspecific protein reactivity^25,26^. ML175 did not perturb the La antigen conformation (see Supplementary Fig. S2), suggesting it may not react indiscriminatingly with protein cysteine thiol residues.

### ML175 protects mice from LPS induced inflammation

ML175 treated bone marrow derived macrophages (BMDMs) showed a dose dependent decrease in the secretion of IL-6 on LPS stimulation (Fig. 7a). This result suggested that ML175 could potentially protect mice against LPS mediated inflammatory shock. Mice treated with 50 mg/kg LPS show a dramatic increase in serum IL-6, and TNFα, within 30 minutes (Fig. 7b,c). This rapid pro-inflammatory response is significantly attenuated in mice treated with ML175 (Fig. 7b,c). In a subsequent proof of principle study, wild type mice pre-treated with a daily *ip* injection of ML175 20 mg/kg for 3 days followed by a single intra peritoneal dose of LPS (15 mg/kg) were found to be less susceptible to LPS induced inflammation as monitored by body temperature (Fig. 7d) and survival (Fig. 7e). The attenuated inflammatory phenotype of ML175 treated mice confirms that ML175 can act as an anti-inflammatory agent by irreversibly binding to GSTO1-1. Further studies could usefully examine the pharmacokinetics of ML175, to optimize its delivery and evaluate specific target engagement in cells and *in vivo*.

## Discussion

In previous studies we have shown that GSTO1-1 deficient macrophages have a defect in their pro-inflammatory response to LPS^6,18^. In this study we characterized GSTO1-1 deficient mice and found that they have significant defects in their response in several distinct inflammatory models. Although unchallenged GSTO1-1 deficient mice appear to be phenotypically normal in most regards, hematological analysis revealed a relative deficiency of monocytes and eosinophils. This could possibly contribute to their abnormal responses to pro-inflammatory stimuli given the role of macrophages in inflammation. The cause of the relative deficiency of circulating monocytes and eosinophils is not immediately obvious but given the effects of GSTO1-1 deficiency on macrophage metabolism^18^ it is possible that this deficiency could affect the differentiation or survival of these particular cell lineages. Noting the many phenotypic similarities between GSTO1-1 deficient mice and mice deficient in TLR4 or MyD88, we considered the possibility that they may also have a similar hematological phenotype. However, there is a paucity of published data on the basic hematology of unchallenged TLR4 or MyD88 deficient mice that limits their comparison with GSTO1-1 deficient mice. The observation that MyD88 deficient mice have impaired monopoiesis during bacterial infection does however suggest that there may be a hematological anomaly that warrants further investigation^27^.

To determine if there was a change in the expression of other GSTs to compensate for the deficiency of GSTO1-1 we examined the expression of GSTs from the Alpha, Mu, Pi, Theta, Zeta, Omega and Kappa classes. Although GSTO2-2 has the greatest structural and functional similarity to GSTO1-1 and is the most likely enzyme to duplicate the functions of GSTO1-1, its expression was unchanged in *Gsto1* ^−/−^ mice. Among the other GSTs evaluated we found decreased expression of GSTA1/2 and GSTM2-2. These GSTs are thought to be up-regulated by oxidative stress via the Nrf2 signaling pathway. Similarly, *Gsto1* ^−/−^ mice had lower expression of NQO1, an enzyme that is also strongly induced by oxidative stress via Nrf2 signaling^28,29^. Thus, there is no evidence to suggest that other GSTs are up regulated to compensate for the GSTO1-1 deficiency and although GSTO1-1 catalyses several reduction reactions, the available data suggest that the deficient mice are not the subject of oxidative stress. This result contrasts with the analysis of gene expression studies of cell lines treated with a GSTO1-1 inhibitor (C1-27) where genes with an Nrf2 signature were significantly up regulated^3^. However, it is likely that the physiological situation induced by genetically determined GSTO1-1 deficiency *in vivo* may differ significantly from that induced transiently by a small molecule inhibitor that may have additional off-target effects^3^.

The resistance of GSTO1-1 deficient mice to LPS mediated inflammatory shock observed in the present studies has confirmed *in vivo* that GSTO1-1 is required for the normal inflammatory response to LPS. GSTO1-1 deficiency diminished the release of pro-inflammatory cytokines IL6, IL-1β and TNFα, and attenuated the acute hepatic pathology that occurs in normal mice exposed to LPS. This phenotype is consistent with the attenuated response of TLR4 and MyD88 deficient mice to LPS^21,22^. The observation that heterozygous mice (*Gsto1* ^+/−^) that express 50% of the normal GSTO1-1 levels are less sensitive to LPS than normal mice suggests that GSTO1-1 inhibitors would not need to completely inhibit GSTO1-1 to be of value in reducing inflammatory responses mediated through TLR4.

The incidence of obesity and type 2 diabetes (T2D) is rapidly increasing and it is a significant global health issue. Insulin resistance, a hallmark of T2D is commonly associated with systemic inflammation that is characterized by the elevated production of IL-1β and the activation of pro-inflammatory signaling pathways^30^. Free fatty acids (FFA) are increased in subjects with T2D and they have been shown to induce inflammatory cytokine production via TLR4 signaling^31,32^. Consequently, in mouse models, the consumption of a high fat diet can result in obesity and high levels of circulating FFA that can induce inflammation and insulin resistance. Multiple studies have reported that TLR4 deficiency attenuates obesity and insulin resistance in mice^32–37^. In the present investigation, we found that GSTO1-1 deficient mice consuming a high fat diet present a similar phenotype to that of TLR4 and MyD88 deficient mice^23,24^ and do not exhibit marked obesity and insulin resistance. Several studies have attributed the macrophage-induced impairment of insulin signaling in T2D to the release of IL-1β^38,39^. Our observation of diminished IL-1β expression in the serum and spleen of GSTO1-1 deficient mice treated with LPS therefore reveals a possible mechanism by which GSTO1-1 deficiency attenuates insulin resistance in mice on a high fat diet. This result again confirms the critical role played by GSTO1-1 in pro-inflammatory signaling and suggests the possibility that GSTO1-1 inhibitors may be of value in the treatment of T2D since IL-1β inhibitors have already been found to be effective in modulating insulin resistance^38,39^.

Since luminal bacterial flora are thought to be an important trigger in the development of inflammatory bowel disease, there have been extensive investigations into the role of TLR4 signaling and the innate immune system in the etiology of colitis (reviewed in^40^) and polymorphisms in NOD2 and TLR4 have been associated with increased susceptibility to Crohn’s disease^41,42^. Deficiency of TLR4 and MyD88 have been shown to sensitize and mice to DSS mediated colitis and previous studies have concluded that TLR4 and MyD88 are required for the control of bacterial translocation and triggering the repair of the intestinal epithelium^43–45^. Our current results with GSTO1-1 deficient mice showing increased clinical severity of colitis, increased escape of bacteria from the colon and increased epithelial cell replication indicate that GSTO1-1 deficient mice follow a similar clinical course in the DSS colitis model to that occurring in TLR4 and MyD88 deficient mice.

Thus, in the models of inflammation that we have investigated, we have consistently observed a phenotype similar to that of TLR4 deficient or MyD88 deficient mice, strongly confirming the view that GSTO1-1 makes a significant contribution to pro-inflammatory signaling via the TLR4 pathway. Previous published studies with the TLR3 ligand Poly IC^6^ and our unpublished studies with ligands such as PAM3CSK4, R848 and CpG have indicated that GSTO1-1 is not required for pro-inflammatory signaling through TLR1/2, TLR3, TLR7 or TLR9.

Our crystallographic study demonstrated that the GSTO1-1 inhibitor ML175 binds in the active site and forms a covalent bond with the sulfur atom of the active site cysteine (C32). The formation of this covalent complex results in the irreversible inactivation of GSTO1-1. In a proof of concept study we found that *ip* injection of ML175 in mice attenuated the LPS induced release of pro-inflammatory cytokines (IL-6, TNFα) and provided significant protection against the inflammatory shock associated hypothermia caused by LPS. This protection was not as profound as that provided by genetic deficiency of GSTO1-1 and may be associated with sub optimal bioavailability and receptor occupancy below 100%, but indicates the clear potential of GSTO1-1 inhibitors as anti-inflammatory agents.

It is possible that ML175 could itself form the basis of drug development. Although it covalently labels its target and therefore necessarily has some degree of protein-reactivity, such a mechanism is not uncommon amongst FDA-approved drugs^46^. However, reactivity in drugs is usually designed late in development or discovered serendipitously^46^ and not inherited from a high throughput screening hit, such as ML175. For this reason, it is important to determine that ML175 is not generally protein-reactive, which is why we determined its propensity for thiol reactivity using the ALARM NMR assay. The negative readout by ML175 in this assay involving highly reactive protein thiols was surprising, but vindicates use of ML175 as a tool for GSTO1-1. Indeed, it has been shown that amongst arrays of electrophilic functional groups, chloromethylamides are relatively less reactive and may be considered reasonable starting points for optimization^47,48^.

The glutathionylation/deglutathionylation of proteins is emerging as a significant mechanism regulating protein function and has been implicated in the regulation of important signaling pathways^11,12,49^. Monocytes and macrophages are key cellular components of the innate immune system and the impact of glutathionylation on macrophage function and inflammation has been recently reviewed^50–52^. Significantly, TNFα release, HIF1α stabilization, STAT3 phosphorylation and Caspase 1 activation in macrophages have all been shown to be regulated by redox potential and glutathionylation^52^. GSTO1-1 catalyses the deglutathionylation of protein thiols and could therefore play a significant role in modulating the glutathionylation of intracellular proteins that participate in specific signaling pathways^10^. The attenuation of the inflammatory response to LPS by the administration of the GSTO1-1 inhibitor ML175 indicates that the enzymatic activity of GSTO1-1 is required for its pro-inflammatory role further suggesting that the glutathionylation of a key protein in the TLR4 pro-inflammatory pathway may act as a regulatory switch.

This study has identified GSTO1-1 as a novel target for the development of drugs for the treatment of inflammation mediated via TLR4 and possibly the treatment of the inflammation associated with obesity. The suitability of GSTO1-1 as a drug target is enhanced by the observation that mice heterozygous for GSTO1-1 deficiency show partial protection against LPS mediated inflammation suggesting that complete pharmacological inhibition of GSTO1-1 may not be required to achieve a significant response. The value of GSTO1-1 as a target is also enhanced by the observation that homozygous GSTO1-1 deficient mice do not exhibit any overt pathology. Thus pharmacologically induced GSTO1-1 deficiency may have few side effects apart from those potentially induced by off target effects of the inhibitor. Importantly the absence of any adverse effects arising from GSTO1-1 deficiency is also apparent in humans. Although several *GSTO1*alleles that encode unstable enzyme variants occur in human populations, no cases of GSTO1-1 deficiency with a deleterious phenotype have been reported^2,53^. Additional benefits to be gained by the use of small molecule inhibitors of GSTO1-1 to regulate the expression of IL-1β include their low cost of production and their potentially short half-life relative to biologics such as canakinumab and rilonacept would allow their rapid withdrawal if a severe infection occurred.

## Methods

### Animals

*Gsto1* knockout mice were supplied by Taconic Biosciences (U.S.A), Model TF1210 Gsto1/NM_010362. The mice were generated by the deletion of a 347 bp fragment containing exons 1 and 2 of the *Gsto1* gene in 129/SvEv ES cells. The ES cells were subsequently injected into C57BL6/albino embryos. The strain was subsequently back crossed to C57BL6 (Charles River) mice and genotyping was undertaken by PCR using primers designed by Taconic. Heterozygotes were mated to generate knockout and wildtype control littermates. In most cases mice were 8 weeks of age when selected for an experiment. Mice were maintained at the ANU Biosciences facility under controlled animal room conditions. The animals were fed regular animal diet and water *ad libitum*. All experiments were undertaken under protocols approved by the ANU Animal Experimentation Ethics Committee according to Guidelines provided by the Australian National Health and Medical Research Committee.

### Reagents and antibodies

All reagents were purchased from Sigma unless indicated otherwise. All GST antibodies were generated in house from recombinant antigens or were gifts from colleagues as previously reported^54^. The GSTO1-1 inhibitor ML175 was prepared by previously described methods^55^.

### Immunoblotting

SDS-PAGE and non-reducing SDS-PAGE were performed as described previously^56^. Separated proteins were transferred onto nitrocellulose membranes^57^ and immunodetected using primary antibodies at a 1:1000 dilution and probed with a goat anti-rabbit IgG horseradish-peroxidase-conjugated secondary antibody (Dako). Chemiluminescence was detected by the ECL Rapid Step chemiluminescence detection system (Calbiochem, Merck, Germany).

### S-(4-nitrophenacyl)glutathione (4-NPG) reduction

GSTO1-1specific activity was determined with 4-NPG as described previously^58^. Briefly, 5 mM 4-NPG was incubated with 75 μg of cell lysate protein in 100 mM Tris, pH8.0, 1.5 mM EDTA and 10 mM β-mercaptoethanol. The reduction of 4-NPG was measured spectrophotometrically at 305 nm at 37 °C and the specific activity was calculated based on an extinction coefficient of 1.1 mM^−1^ cm^−1^.

### *In vivo* temperature measurements

Mouse body temperature was determined with temperature-recording probes (DAS-7009 smart reader, BMDS) that were implanted sub-cutaneously oriented longitudinally above the shoulder 24 hours prior to LPS injections. The body temperature was monitored over 24 hours to confirm that no infection occurred due to the insertion of the probe. For LPS experiments, body temperature of less than 24 °C was considered as an ethical surrogate marker of death and mice were euthanized immediately by CO_2_ asphyxiation in accordance with the ANU animal ethics protocol.

### Organ collection and homogenization

Organs were harvested in assay specific buffers. For immunoblots and biochemical assays, tissues were dissected, washed in PBS and homogenized in T-PER (Thermo Scientific) using an Ultraturux homogenizer. Homogenates were centrifuged at 20000 × g at 4 °C to remove tissue debris and stored at −20 °C until further use.

### Hematology

Blood was collected into heparin-coated sterile tubes by retro-orbital bleeding from 8 week old mice. Differential cell counts were immediately analysed using an ADVIA Haematology systems instrument (Siemens). Hemoglobin determinations in the colitis model were undertaken on blood samples collected in EDTA coated tubes. Briefly, blood was added to Drabkin’s solution (100 mg/L sodium cyanide, 300 mg/L of potassium ferricyanide) in a 1:20 ratio and absorbance was read at 540 nm after 5 minutes and compared with a standard curve.

### Real time RT-PCR

Total RNA was extracted from cells and tissues in trizol as per manufacturer’s instructions (Invitrogen). Trizol-lysed samples were incubated for 5 minutes at room temperature and passed repeatedly through a 25-gauge syringe/needle to enhance cell lysis. 200 μl of chloroform was added to each sample and the tube was shaken vigorously for 30 seconds. The samples were centrifuged at 15000 × g for 15 minutes at 4 °C and the top clear layer was transferred to a fresh tube followed by the addition of 600 μl of 70% ethanol. The samples were then transferred to RNA spin columns and RNA was isolated as per manufacturer’s instructions (Qiagen RNA extraction kit). The extracted RNA was treated with DNase to remove genomic DNA contamination (Ambion DNAse kit) and cDNA was synthesized with an Invitrogen First strand cDNA synthesis kit. Real time PCR was carried out on an ABI 7900HT thermocycler and relative transcript levels were calculated by the ΔΔCt method using GAPDH as an internal control to which all transcripts were normalized.

### Injections and treatments

Indicated doses of LPS (Sigma) (5-50 mg/kg) were intra-peritoneally injected. For inflammatory models, mice were fed a high fat diet (23% fat, 0.1% carbohydrates, cholesterol pellets) (Specialty Feeds) for indicated time periods. For the induction of inflammatory bowel disease, mice received 3% dextran sodium sulphate (DSS) (molecular mass 50KDa, MP Pharmaceuticals) in their drinking water for 5 consecutive days followed by regular drinking water for 3 days. DSS/water was replaced with fresh DSS on Day 3.

Mice were treated with the GSTO1-1 inhibitor ML175 (10 mg/kg in ethanol/Cremophore EL 1:1 at −48, −24, and −2 hours prior to ip LPS injection (15 mg/kg). Cells treated with varied concentrations of ML175 in DMSO (0.25 μM- 5 μM) where indicated for 2 hours prior to LPS stimulation.

### Glucose tolerance test (GTT) and Insulin tolerance test (ITT)

GTT and ITT were performed after 12 and 13 weeks on HFD respectively. For GTT, the mice were fasted for 16 hours and then injected intra-peritoneally with 1 g/kg glucose solution. For ITT, the mice were fasted for 6 hours and then injected with 1 IU/kg insulin intra-peritoneally. Blood glucose levels were determined at indicated time points using a FreeStyle Optium Xceed blood glucose meter.

### Isolation and culture of primary adipocytes

Adipose tissue (3 g) was finely minced into sterile tubes and incubated for 1 hour at 37 °C in DMEM supplemented with 25 mm HEPES, 4 mg/ml collagenase type II, and 40 mg/ml albumin. Digested tissues were filtered through nylon mesh under sterile conditions and the cell suspension was centrifuged at 100 rpm for 5 minutes. The floating cells (upper pale layer) were collected and washed in DMEM supplemented with 10 mm HEPES, 20% FBS, 1% BSA and penicillin/streptomycin/neomycin. 10^5^ cells were seeded in 6-well plates and covered with a coverslip and cultured for 10 days at 37 °C, 5%CO_2_. The lipid content in cells was quantified by staining with Oil Red O. Cells were fixed with 10% formaldehyde for 10 minutes at room temperature followed by washes in 60% isopropanol. Cells were stained with 0.2% oil red O staining solution for 10 minutes at room temperature followed by four washes in distilled water. The stain was eluted by incubating the cells in absolute isopropanol for 10 minutes. Supernatants were collected and absorbance was measured at 500 nm.

### Flow cytometry

Adipose tissue was prepared as above. Cells were washed three times in PBS/1% FBS and blocked with FCblock followed by staining with F4/80-APC and Propidium iodide for 30 minutes at 4 °C. Cell preparations were analysed on a FACS Calibur.

### Clinical score

Clinical scores were assigned to DSS treated mice based on body weight, presence of faecal occult blood, stool consistency and blood in orifice which were determined daily for 8 days. Scoring was performed blind by an independent observer.

*Weight loss:* No weight loss was given a score of 0, a loss of 1% from baseline (weight on Day 0) was a 1, loss of 5–10% was a 2, loss of 10–20% was a 3 and a loss of >20% as a 4. *Bleeding from orifice*: No presence of blood at the anal orifice was scored as 0, mild redness as 1, positive for occult blood as 2, mild bleeding as 3 and gross bleeding as 4. *Stool consistency*: Normal, well-formed pellets were scored as 0, pasty pellets as 1, pasty and semi-formed pellets that do not adhere to the anus as 2, pasty and semi-formed pellets that do adhere to the anus as 3 and liquid stool that adheres to the anus as 4. *Faecal occult blood*: Blood in the faeces was determined daily using a ColoScreen Occult Blood Test (Helena Laboratories). A score of 1 was assigned if the screen turned pale blue and a score of 2 if the screen turned deep blue.

### Isolation of mesenteric nodes and culture of gut flora

Mesenteric nodes were dissected into sterile PBS and homogenized using a micro pestle under sterile conditions. Cells were suspended in sterile Luria broth (LB) without antibiotic selection and plated on LB plates overnight (12 hours) at a 10^6^ dilution. Colonies were counted by naked eye and scored as number of colonies/ml of cell suspension.

### Proliferation assay

Proliferation of intestinal epithelial cells at the proximal, mid and distal sections of the colon was measured using a 5-bromo-2′-deoxyuridine (BrdU) Staining Kit (Life technologies). Mice were injected intraperitoneally with BrdU (10 ml/kg) 2 hours prior to harvesting organs. BrdU was detected immunohistochemically on formalin fixed colon sections according to the manufacturer’s instructions. Images were captured using an Olympus IX71 inverted brightfield/fluorescence microscope. PCNA and c-myc were quantified by immuno blotting.

### Tissue pathology

All histopathology was undertaken blind by an independent pathologist. Following fixation in 10% neutral buffered formalin the tissues were processed into paraffin blocks using routine laboratory methods and 4 µm sections examined following routine hematoxylin-eosin (H&E) staining.

To examine the progression of the pathology associated with LPS mediated inflammation in *Gsto1* ^+/+^ and *Gsto1* ^−/−^ mice were injected with 50 mg/kg LPS and euthanized at different time points (0.5, 1, 3, 6 and 12 hours) N = 3/time point. The liver and the spleen were examined with and without LPS treatment. The type and location of inflammatory cells were assessed. The amount of inflammation, necrosis and steatosis in the liver was graded as absent, mild, moderate or severe. The number of tingable body macrophages and neutrophils in the spleen was graded as absent, mild, moderate or marked increase.

The liver and adipose tissue were examined following a high fat diet for 13 weeks. The type and location of inflammatory cells were assessed. The amount of inflammation and steatosis in the liver was graded as absent, mild, moderate or severe.

Colons were examined following DSS treatment for 5 days. Following fixation the preparation of 4 µm H&E stained sections as described above, the type and location of inflammatory cells (colitis) was assessed by microscopy. The severity of the acute inflammation was graded as absent, mild, moderate (presence of mucosal erosions) or severe (presence of mucosal ulceration). The amount of chronic inflammation was graded as absent, mild, moderate or severe.

### Cytokine measurement

The blood and the lungs were collected for cytokine measurement. The plasma was collected from the red blood cells by ultracentrifugation (16,000 rpm for 10 minutes). The lungs were mashed through a 70 µm cell strainer (BD Biosciences) and the supernatant collected into PBS. The level of IL-1ß, IL-6, and TNF-α were measured using a mouse ELISA kit (Elisakits.com, Australia) according to the manufacturer instructions.

### Crystal structure of GSTO1-1with ML175

GSTO1-1 was crystallized as described previously^1^. Grains of solid compound ML175 was seeded into a drop containing crystals and allowed to equilibrate for 2 weeks. Crystals were transferred to a cryoprotectant solution comprised of 50 mM sodium acetate pH 4.6, and 1.0–1.2 M (NH_4_)_2_SO_4_ and 5% glycerol prior to flash-cooling to 100 K. X-ray data were collected to 2.25 Å resolution using a Rigaku 007 HF X-ray Generator producing CuKα X-rays with Varimax optics. X-ray diffraction patterns were collected using a Mar345 desktop beamline. Diffraction data were integrated, merged and scaled with the HKL2000 package^59^. The structure was solved by PHASER^60^, using the structure of GSTO1-1 in complex with GSH (PDB ID 1EEM). Iterative cycles of model building and refinement were performed in COOT^61^ and REFMAC5^62^. Electron density in 2mFo-DFc and mFo-DFc electron density maps was interpreted as bound ML175 and included in the model. X-ray data and model quality are given in Supplementary Table 1 and the coordinates for the present structure have been submitted to the protein data bank (ID 5V3Q).

### ALARM NMR Assay

In brief, test compounds (400 μM final concentration) were incubated with ^13^C-methyl-labelled La antigen (50 μM final concentration) at 37 °C for 1 h and then 30 °C for 15 h prior to data collection. Each compound was tested in the absence and presence of 20 mM DTT. Data were normalized to DMSO vehicle control. Data were recorded at 25 °C on a Bruker 700 MHz NMR spectrometer equipped with a cryoprobe (Bruker) and autosampler. Samples were acquired with 16 scans, 2048 complex points in F2, and 80 points in F1 using standard protein HMQC and water suppression pulse sequences. Nonreactive compounds were identified by the absence of chemical shifts (^13^C-methyl) independent of the presence of DTT. Reactive compounds were identified by characteristic chemical shifts and peak attenuations in the absence of DTT^25,26^.

### Statistical analysis

Data were expressed as the mean ± standard error and analysed using Prism 4 (Graphpad software Inc.). Statistical significance was calculated by standard t-tests or by Mann-Witney non parametric tests between *Gsto1* ^−/−^ and WT groups. ANOVA with Tukey’s test was used where comparisons between multiple groups/treatments were made. For Kaplan Meier survival curves P values were determined by the Gehan-Breslo-Wilcox test comparing two curves at a time. P values were then adjusted for multiple comparisons by the Holm-Sidak method. All experiments were performed in triplicate unless otherwise stated.

## Electronic supplementary material

Supplementary information
