# Supplementary material for: GSTO1-1 plays a pro-inflammatory role in models of inflammation, colitis and obesity
Source: Sci Rep. 2017 Dec 19;7:17832. doi: 10.1038/s41598-017-17861-6 (PMC5736720; doi:10.1038/s41598-017-17861-6)
Supplement: Supplementary file 1 — Supplementary information [file 41598_2017_17861_MOESM1_ESM.pdf]

Supplementary Data

GSTO1-1 plays a pro-inflammatory role in models of inflammation, colitis and obesity

Deepthi Menon[1,2], Ashlee Innes[1], Aaron J Oakley[3], Jane E Dahlstrom[4], Lora M. Jensen[1], Anne Brüstle[1], Padmaja Tummala[1], Melissa Rooke[1], Marco G Casarotto[1], Jonathan B. Baell[5,8], Nghi Nguyen[5], Yiyue Xie[5], Matthew Cuellar[6], Jessica Strasser[6], Jayme L Dahlin[7], Michael A. Walters[6], Gaetan Burgio[1], Luke A J O'Neill[2] and Philip G Board[1].

[1]John Curtin School of Medical Research, Australian National University, Canberra, ACT 2600, Australia.
[2]School of Biochemistry and Immunology, Trinity Biomedical Sciences Institute, Trinity College Dublin, Dublin 2, Ireland.
[3]School of Chemistry, University of Wollongong, Wollongong, NSW 2522, Australia
[4]ACT Pathology and ANU Medical School, The Canberra Hospital, Australian Capital Territory, Australia.
[5]Monash Institute of Pharmaceutical Sciences, Monash University, Parkville, Victoria 3052, Australia.
[6] Institute for Therapeutics Discovery and Development, University of Minnesota, Minneapolis, MN, USA
[7] Department of Pathology, Brigham and Women's Hospital, Boston, MA, USA
[8] School of Pharmaceutical Sciences, Nanjing Tech University, Nanjing 211816, People's Republic of China

SUPPLEMENTARY FIGURES

Supplementary Figure S1

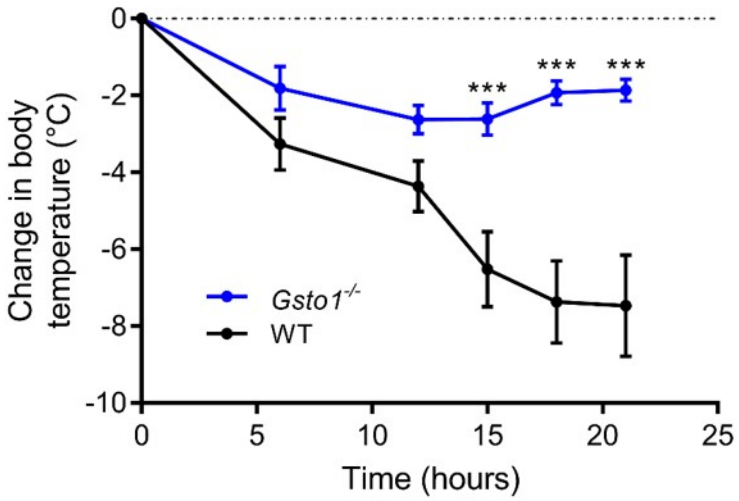

Supplementary Figure 1: Change in body temperature of female mice treated with LPS 7.5mg/kg. Mean ± SE n=6. ***P<0.001.

Supplementary Figure S2

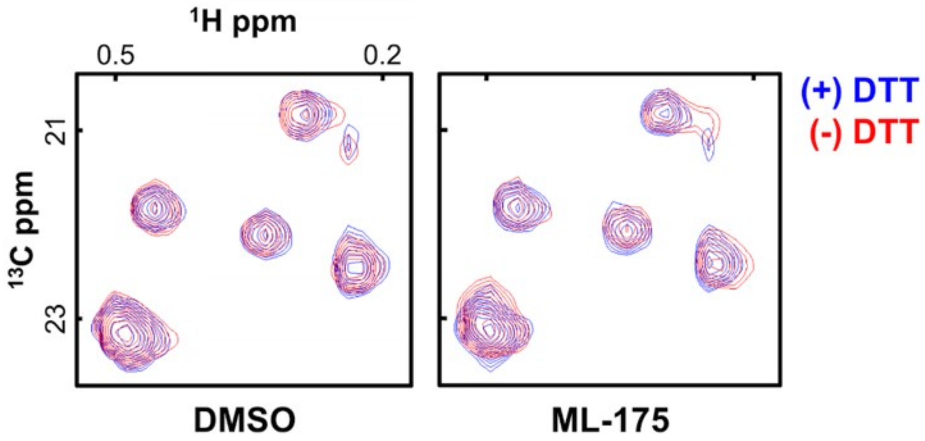

Supplementary Figure 2. ML175 does not perturb the La antigen conformation by ALARM NMR counter-screen for non-specific thiol reactivity. Shown are ^{1}H-^{13}C HMQC spectra of key ^{13}C-labeled methyl groups of the La antigen after incubation with either DMSO or ML175. ML-175 was incubated with the La antigen probe in the presence (blue spectra) and absence (red spectra) of excess DTT. Data are normalized to DMSO vehicle control. ALARM NMR-positive compounds perturb the La antigen, as evidenced by pronounced significant peak shifts and/or peak signal attenuations in the absence of DTT.

Supplementary Table 1. Crystallographic data for the GSTO1-1 complex with ML175.

**X-ray data**	
Space Group	$P3_121$
Unit Cell Parameters	a = 57.102, b = 57.102, c = 139.6 Å; α = β = 90° γ = 120°
Resolution range (Å)	50–2.25 (2.33–2.25)[a]
Total no. of Observations	68,120
No. of unique reflections	12,979
$I/\sigma I$	57.84 (13.51)
R_{merge} (%)	2.7 (13.0)
R_{meas} (%)	3.0 (14.4)
CC1/2 (%)	0.997 (0.991)
Completeness	98.4 (97.4)
Multiplicity	5.2 (5.3)
**Refinement statistics**	
Resolution range (Å)	50–2.25 (2.31–2.25)
No. of reflections (R_{work} set)	11,591 (852)
No. of reflections (R_{free} set)	686 (61)
R_{work} (%)[c]	17.1 (19.3)
R_{free} (%)	23.1 (26.6)
No. of atoms	2,112
⟨B⟩ of structure (Å^2)	24.5
r.m.s.d. from ideal geometry:	
Bond lengths (Å)	0.023
Bond angles (°)	2.227
Chiral Centers (Å^3)	0.129
General planes (Å)	0.011
